# Papillary Thyroid Carcinoma: Molecular Distinction by MicroRNA Profiling

**DOI:** 10.3389/fendo.2022.834075

**Published:** 2022-02-23

**Authors:** Francesca Galuppini, Simona Censi, Isabella Merante Boschin, Matteo Fassan, Marta Sbaraglia, Nicola Valeri, Jens Claus Hahne, Loris Bertazza, Giada Munari, Marco Galasso, Luciano Cascione, Susi Barollo, Massimo Rugge, Federica Vianello, Angelo Paolo Dei Tos, Caterina Mian, Gianmaria Pennelli

**Affiliations:** ^1^ Pathology Unit, Department of Medicine (DIMED), University of Padua, Padua, Italy; ^2^ Endocrinology Unit, Department of Medicine (DIMED), University of Padua, Padua, Italy; ^3^ Veneto Institute of Oncology, Istituto Di Ricovero E Cura a Carattere Scientifico (IRCCS), Padua, Italy; ^4^ Division of Molecular Pathology, The Institute of Cancer Research, London, United Kingdom; ^5^ Laboratorio per le Tecnologie delle Terapie Avanzate (LTTA), Department of Morphology, Surgery and Experimental Medicine, University of Ferrara, Ferrara, Italy; ^6^ Bioinformatics Core Unit, Institute of Oncology Research, Bellinzona, Switzerland; ^7^ Department of Radiotherapy, Veneto Institute of Oncology, Istituto Di Ricovero E Cura a Carattere Scientifico (IRCCS), Padua, Italy

**Keywords:** microRNA, papillary thyroid cancer, histological variant, miR-205, miR-146, miR-21, hobnail, *BRAF*

## Abstract

Papillary thyroid carcinoma (PTC) is a miscellaneous disease with a variety of histological variants, each with its own mutational profile, and clinical and prognostic characteristics. Identification of microRNA (miRNA) expression profiles represents an important benchmark for understanding the molecular mechanisms underlying the biological behavior of these unique PTC subtypes in order that they be better characterized. We considered a series of 35 PTC samples with a histological diagnosis of either hobnail (17 cases) or classical variant (nine cases) and with a specific *BRAF* p.K601E mutation (nine cases). We determined the overall miRNA expression profile with NanoString technology, and both quantitative reverse transcription–PCR and *in situ* hybridization were used to confirm selected miRNAs. The miRNA signature was found to consistently differentiate specific histotypes and mutational profiles. In contrast to the *BRAF* p.K601E mutation and classic PTCs, three miRNAs (miR-21-5p, miR-146b-5p, and miR-205-5p) were substantially overexpressed in the hobnail variant. The current study found that different miRNA signature profiles were linked to unique histological variants and *BRAF* mutations in PTC. Further studies focusing on the downstream pathogenetic functions of mRNAs in thyroid neoplasms are warranted.

## Introduction

Papillary thyroid carcinoma (PTC) is the most common thyroid malignancy, accounting for 80%–90% of all tumors affecting this gland, and predominantly affects women (1, 2). Despite the prognosis of PTC being excellent (10-year survival is up to 90%) (3), a rise in mortality has been mainly linked to local tumor persistence or recurrence (10-year survival is from 49% to 68%) or distant metastases (10-year survival between 25% and 42%) (3). Therefore, it is fundamental to identify those patients who have an increased risk of recurrence and, therefore, who need more extensive surgery or a targeted postsurgical follow-up. Several factors have already been identified to be prognostic in the assessment of tumor aggressiveness such as age, gender, tumor size, pTNM, stage, and extrathyroid extension. Recently, of these prognostic factors, interest has focused on two particular variables: histological variants and molecular features.

In addition to the classic morphology of PTC, more than 10 different histologic variants have been described. Some of these can be distinguished solely based on a peculiar microscopic appearance, whereas others appear to have distinct clinical and prognostic characteristics. The latter group includes the follicular variant, characterized by a favorable prognosis (to the point where some capsulated variants have been renamed noninvasive follicular thyroid neoplasm with papillary-like nuclear features or “NIFTP” (4)). Other variants have been characterized by more aggressive behavior: tall cells, columnar, and hobnail. In particular, hobnail PTC (HoV-PTC) has been recently identified as a rare but aggressive histological variant (about 0.2% of all PTCs). It typically is found at an advanced stage at the time of diagnosis and tends to develop in lymph nodes as distant metastases. The hobnail variant shows a significant mortality rate, and its disease-specific survival rate is approximately 46%–66% (5–7). *BRAF* p.V600E is the most common mutation in these tumors, which is present in 50%–65% of HoV-PTC cases, followed by *TP53* (56%). Recent studies have also reported *RET/PTC1* rearrangements and *TERT* promoter mutations in HoV-PTC (8, 9). Because of its low incidence and recent identification, further studies are needed to clearly define this variant from molecular, histological, and clinical points of view.

To date, with regard to molecular features of thyroid tumors, *BRAF* and *TERT* promoters have been the genes most investigated as possible outcome indicators. *BRAF* p.V600E mutations are considered reliable preoperative prognostic factors with regard to optimizing the choice of surgical treatment. In fact, this mutation is very frequent in PTC, varying from 14% to 64% of cases (10). *BRAF* p.V600E is more frequent in classic and tall-cell variants (11), while the p.K601E mutation is peculiar to the follicular variant (*BRAF* p.K601E FV-PTC), and seems to be a favorable prognostic factor (12). Although numerous studies have correlated the p.V600E mutation with well-known prognostic factors, such as extrathyroid extension and advanced stages at diagnosis, a significant correlation with an outcome has not yet been demonstrated (13). Therefore, the identification of a new marker associated with *BRAF*-mutated tumors and a poor prognosis is required and may be used as a new therapeutic target and follow-up marker in PTC.

In response to this necessity, a new class of molecules has been studied during the last decade: microRNAs (miRNAs). MiRNAs are small noncoding single-stranded RNA filaments that act as negative regulators of gene expression at the posttranscriptional level. MiRNAs bind the untranslated region (UTR) of target mRNAs and block their translation or degradation, regulating the downstream cascade (14, 15). The role of miRNAs has therefore proved to be crucial when researching regulatory mechanisms of the main genes involved in neoplastic development, becoming both therapeutic targets and diagnostic and prognostic markers. In recent literature, several miRNAs, including miR-222, miR-221, and miR-21, have been found to be overexpressed in PTC tissues compared to normal thyroid tissue (16). These results suggest that distinct miRNA profiles are correlated with PTC tumorigenesis, progression, and prognosis (17).

In light of these data, we wanted to undertake a pilot study to test whether there was an association between rare histological variants of PTC with more aggressive clinicopathological features or conversely with more benign course between core genetic mutations of PTC and between miRNA signatures. For these reasons, we decided to trace the miRNA expression profiles of representative samples of p.K601E-PTC, hobnail, and classic variants of PTC, which had been previously investigated for *BRAF* p.V600E, with the aim of identifying new markers associated with the characteristic biological behavior of this type of carcinoma.

## Materials and Methods

### Study Population

All patients underwent a total thyroidectomy or lobectomy between 2008 and 2016 at the University Hospital of Padua, with a histological diagnosis of PTC. Patient material was collected from the archives of the Surgical Pathology Unit at Padua University Hospital (Padua, Italy). Hematoxylin and eosin-stained slides from formalin-fixed paraffin-embedded tissues were reviewed by two pathologists (GP and FG).

All cases of HoV-PTC and p.K601E-PTC were selected. Nine CV-PTC cases were included as a control cohort. In all patients, a fine-needle aspiration biopsy was performed on a suspicious node at each ultrasound examination and part of the biopsied material was used for the molecular evaluation of the mutational status of *BRAF*.

All patients involved in the study gave their written informed consent, and the study was approved by the Center’s Ethics Committee of the University Hospital of Padua. All experiments were performed in accordance with relevant guidelines and regulations.

### *BRAF* Mutational Profiling

The genetic material necessary for the molecular analysis of the *BRAF* gene was extracted from the primary tumor of all study patients. Since each sample was initially fixed in formalin and embedded in paraffin for storage, a deparaffining process was carried out for DNA extraction using a “DNeasy Blood & Tissue Handbook” according to the manufacturer’s instructions. After this procedure, qualitative control of the extracted DNA and the determination of its concentration were performed.

### DNA Amplification by PCR and Sequencing

Once the DNA was extracted, the specific sequences encoding the gene under study were amplified by PCR. Each amplification reaction was verified by electrophoresis through a 2% (P/V) agarose gel in Tris-acetate-EDTA (50×). Using Millipore Microcon columns (centrifugal filter device), the amplification products were separated from primers, proteins, salts, and resins. Direct sequencing of the aforementioned exons was performed using a Big-dye Terminator protocol according to the manufacturer’s instructions. Subsequently, each product was purified using Autoseq G-50 Dye Terminator Removal Kit (GE Healthcare, Little Chalfont, UK) columns and then processed by an automatic sequencer ABI PRISM (Applied Biosystems, Bedford, MA, USA).

### NanoString nCounter^®^ and Bioinformatics Analysis

Total RNA extraction was performed using an Ambion Recover All Isolation Kit (Life Technologies, Carlsbad, CA, USA), according to the manufacturer’s instructions. The purity and quality of the extracted RNA were determined by Bio-Analyser (Agilent Technologies, Santa Clara, CA, USA).

MiRNA expression was analyzed using an nCounter^®^ Human v2 miR Expression Assay Kit (NanoString, Seattle, WA, USA) as previously described (18).

This assay detects 800 endogenous miRNAs, five housekeeping transcripts plus six positive and six negative controls. About 150 ng of each total RNA sample was used as input into the nCounter Human miRNA sample preparation. Hybridization was conducted for 16 h at 65°C. Subsequently, the strip tubes were placed into the nCounter Prep Station for automated sample purification and subsequent reporter capture. Each sample was scanned for 555 fields of view on an nCounter^®^ Digital Analyzer (NanoString). Data were extracted using an nCounter^®^ RCC Collector (NanoString). Six samples (three HoV-PTC and three p.K601E-PTC) failed quality control and were excluded from further analysis. Raw data, which were proportional to copy number, were log-transformed and normalized by the quantile method after application of a manufacturer-supplied correction factor for several miRNAs (19). Data were filtered to exclude relatively invariant features (interquartile range = 0.5) and features below the detection threshold defined for each sample by a cutoff corresponding to Δ2 standard deviations of negative control probes plus their mean in at least half of the samples. After the preprocessing steps described above, we plotted relative differences in transcriptional profile between the samples using a multidimensional scaling plot. Using R/Bioconductor and the filtered dataset, limma data analysis were employed with a contrast matrix for the comparisons of interest (19, 20). *P*-values were used to rank miRNAs of interest, and a correction for multiple comparisons was performed using the Benjamini–Hochberg method (21). Raw data that were above background, as well as the corresponding quantile-normalized data, were imported into MultiExperiment Viewer (http://mev.tm4.org/) for visual inspection. Only miRNAs with a *P*-value <0.01 were included in heatmaps. The color red indicates the strong expression of a miRNA, whereas a green point mirrors a reduced level of a determined miRNA.

### Quantitative Real-Time Polymerase Chain Reaction Analysis

Expression of three of the most important dysregulated miRNAs detected using NanoString nCounter^®^ Analysis (hsa-miR-21-5p, hsa-miR-146b-5p, hsa-miR-205-5p) was investigated by quantitative reverse transcription (qRT)–PCR.

For qRT–PCR analysis, an NCodeTM miRNA qRT–PCR kit was used (Invitrogen, Carlsbad, CA, USA) according to the manufacturer’s instructions with the following primers:

- hsa-miR-21-5p (primer: 5′-cgg tag ctt atc aga ctg atg ttg a-3′)

- hsa-miR-146b-5p (primer: 5′-ggg tga gaa ctg aat tcc a-3′)

- hsa-miR-205-5p (primer: 5′-ggt cct tca ttc cac cg-3′)

Results were normalized relative to the values of *RNU6B* nuclear RNA expression (5′-gtt ggc tct ggt gca gg gcc tcc gag gta ttc gca-3′). Reactions were performed in a LightCycler 480 Real Time PCR cycler System (Roche, Milan, Italy). All qRT–PCR reactions were conducted in duplicate, and the results were calculated according to the comparative CT method. This method uses the equation 2^-dCt^, where Ct is the threshold cycle (i.e., the number of cycles at which the fluorescence generated in one reaction exceeds a given threshold), and dCt is the difference between the mean Ct of the gene sample and the mean Ct of the *RNU6B* probe.

### MiR-21 and MiR-205 *In Situ* Hybridization Analysis

*In situ* hybridization (ISH) was conducted on tissues of all samples under examination. The ISH was performed using a signal amplification system (GenPointTMCatalyzed Signal Amplification System; DakoCytomation, Carpinteria, CA, USA) following the protocol provided by the manufacturer and modifications made by Yamamichi et al. (22).

The sections on slides were incubated at 60°C for 30 min, deparaffinized, and subsequently treated with proteinase K (DakoCytomation) for 30 min at room temperature. After gentle rinsing in distilled water and immersion in a 95% ethanol solution for 10 s, sections were air-dried. They were then prehybridized at 49°C–56°C for 1 h with RNA ISH buffer (Ambion, Carlsbad, CA, USA). They were subsequently incubated overnight at 49°C–56°C in RNA ISH buffer containing miRCURYTM LNA probes and labeled with biotin for the detection of hsa-miR-21 (Exiqon, Woburn, MA, USA). As a control, a probe (*U6*, Exiqon) was used at a final concentration of 200 nM. The slides were then washed in TBST and then GenPointTM stringent wash solution (at 54°C for 30 min). The slides were exposed to a fixative containing H_2_O_2_ (DakoCytomation) for 20 min and then a further solution fixative (DakoCytomation) for 30 min. The slides were then exposed to primary streptavidin-horseradish peroxidase antibody, a biotinylated tiamide, a streptavidin-labeled secondary antibody, and a solution chromogen nitroblue tetrazolium, according to the manufacturer’s instructions. The sections were then mildly counterstained with hematoxylin and washed with TBST and water before assembly.

### Statistical Analysis

The *t*-test was used to assess the differences in miRNA expression levels, obtained by qRT–PCR, between Hobnail variant–PTCs, *BRAF* K601E mutation FV-PTCs, and classic variant–PTCs or in different subgroups classified by *BRAF* V600E status. Statistical analyses were performed using MedCalc Statistical Software version 16.4.3 (MedCalc Software bvba, Ostend, Belgium; https://www.medcalc.org; 2016). A *P* < 0.05 was considered statistically significant.

## Results

### Clinicopathological Features

**Figure 1:**
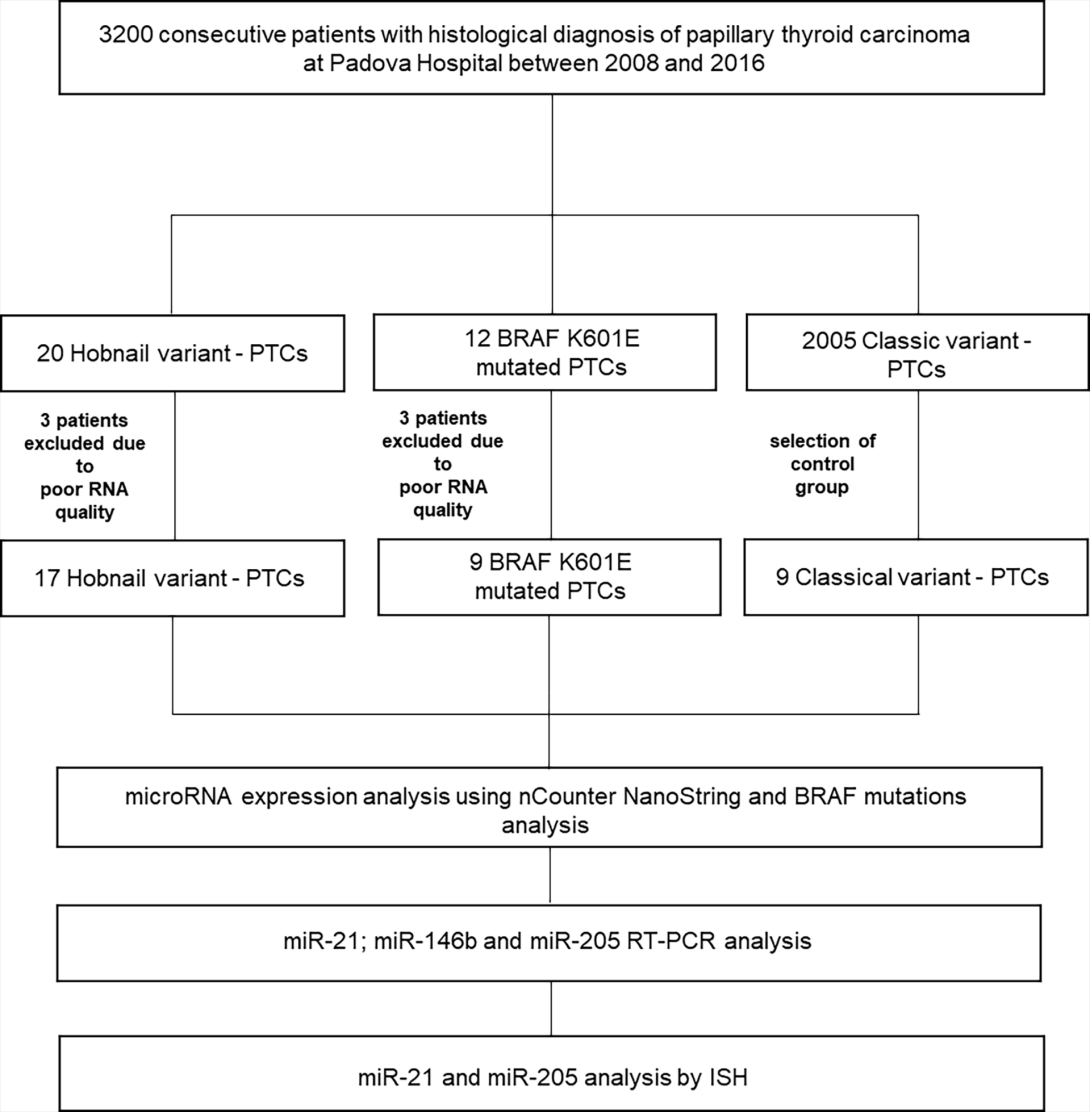
Overview of the study. ISH, *in situ* hybridization; PTC, papillary thyroid carcinoma; RT–PCR, reverse transcription–PCR.

Of the original 32 patients recruited with a histological and molecular diagnosis of HoV-PTC and p.K601E-PTC, 26 patients were included in our study (six patients were excluded from the analysis, and details are shown in **Figure 1**). Nine classic variant (CV)-PTC cases were included as a control cohort. All cases of p.K601E-PTC resulted in a follicular variant (*BRAF* p.K601E FV-PTC). Clinicopathological features are summarized in **Table 1**.

**Table 1:** Clinicopathological features of patients included in this study.

Patient	Age at diagnosis (years)	Gender	Tumor size (cm)	Histological variant	t	n	m	Stage	*BRAF* status
**1**	52	F	1.7	Hobnail	1b	0	0	I	wt
**2**	24	M	4.3	Hobnail	4a	1b	0	I	wt
**3**	46	M	1.2	Hobnail	1b	0	0	I	wt
**4**	38	M	2.2	Hobnail	2	1a	0	I	wt
**5**	48	F	2.4	Hobnail	3b(m)	1b	0	I	wt
**6**	48	M	3.8	Hobnail	3	X	0	I	wt
**7**	63	M	n.v.	Hobnail	4a(m)	1a	0	IVA	wt
**8**	73	F	2.1	Hobnail	2	0	0	I	wt
**9**	56	F	4.5	Hobnail	3b(m)	1a	0	II	wt
**10**	36	F	1.6	Hobnail	1b	1a	0	I	V600E
**11**	65	F	3.7	Hobnail	3a(m)	1a	0	II	V600E
**12**	38	F	1.1	Hobnail	3a(m)	0	0	I	V600E
**13**	29	F	4.1	Hobnail	3a	1a	0	I	V600E
**14**	38	M	2.3	Hobnail	3a(m)	1a	0	I	V600E
**15**	55	M	2.9	Hobnail	3a(m)	1a	0	II	V600E
**16**	32	F	3.0	Hobnail	2(m)	0	0	I	V600E
**17**	69	F	1.8	Hobnail	1b(m)	1a	0	II	V600E
**18**	53	F	1.0	Follicular	1b(m)	0	0	I	K601E
**19**	78	M	8.0	Follicular	3a	X	0	II	K601E
**20**	34	M	1.2	Follicular	1b	0	0	I	K601E
**21**	29	F	3.7	Follicular	2	X	0	I	K601E
**22**	78	F	0.7	Follicular	1a(m)	0	0	I	K601E
**23**	45	F	0.6	Follicular	1a(m)	0	0	I	K601E
**24**	43	F	3.0	Follicular	2	X	0	I	K601E
**25**	42	F	4.5	Follicular	3	X	0	I	K601E
**26**	34	F	2.1	Follicular	2	0	0	I	K601E
**27**	52	F	1.3	Classical	1b(m)	1a	0	I	V600E
**28**	38	F	1.2	Classical	3b(m)	1a	0	I	V600E
**29**	23	F	2.8	Classical	3b(m)	0	0	I	V600E
**30**	35	F	1.5	Classical	1b	1a	0	I	V600E
**31**	61	F	1.1	Classical	1b	0	0	I	wt
**32**	25	F	1.7	Classical	1b(m)	0	0	I	wt
**33**	27	F	1.5	Classical	1b	0	0	I	wt
**34**	43	F	2.2	Classical	2(m)	0	0	I	wt
**35**	54	F	3.0	Classical	2	1a	0	I	wt

M, male; F, female; wt, wild-type.

### *BRAF* p.V600E Mutation Analysis

A *BRAF* p.V600E mutation was found in 8/17 (47%) of HoV-PTC cases and in 4/9 (44%) of CV-PTC cases. A p.V600E mutation was not found in any patient with p.K601E, as previously detected in cytological specimens. All *BRAF* p.K601E mutations were identified in histological samples.

### Identification of MiRNAs Associated With Histological and Mutational Features

In order to define miRNAs that distinguished between patients with characteristic histological variants and mutational profiles, we performed miRNA expression analysis in a cohort of 17 HoV-PTC cases, nine *BRAF* p.K601E FV-PTC cases, and in a randomly selected cohort of nine CV-PTC cases.

**Figure 2:**
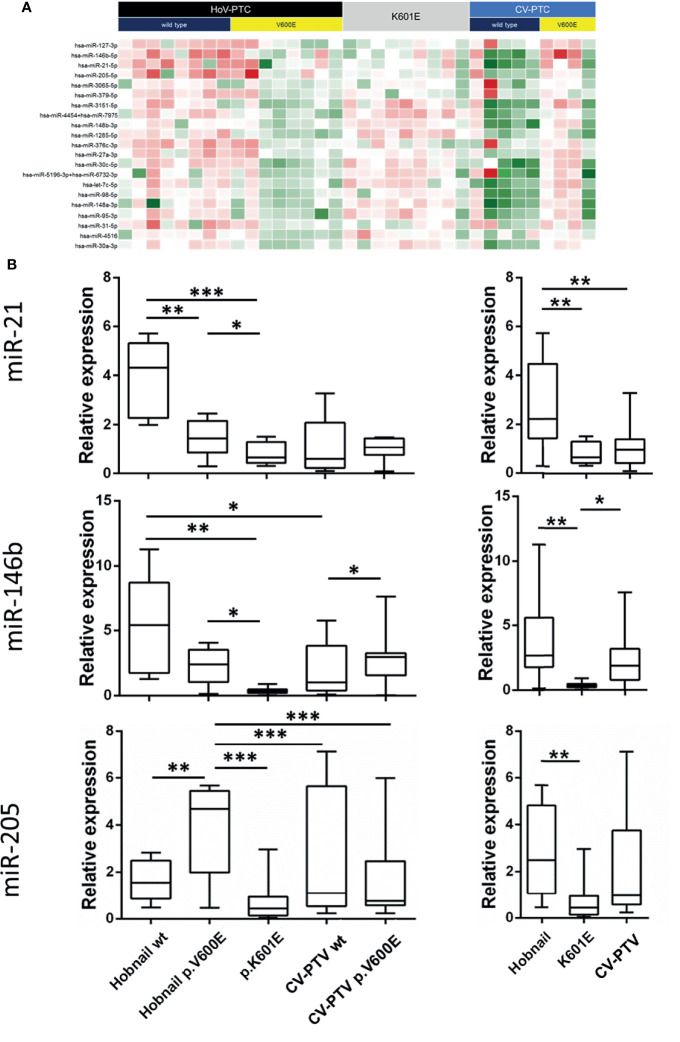
**(A, B)** Heatmap showing miRNA expression in all PTC subgroups. The miRs with a *P*-value <0.01 are shown **(A)**. Plot showing miR-21-5p, miR-146b-5p, and miR-205-5p expression in subgroups divided for a *BRAF* p.V600E mutation (left panel) and in hobnail papillary thyroid carcinoma (HoV-PTC) vs. *BRAF* p.K601E FV-PTC and classic variant (CV)-PTC (right panel). All qRT–PCR reactions were conducted in duplicate, and the results were calculated according to the comparative CT method. **(B)** wt, wild-type. *P < 0.01, **P < 0.001, ***P < 0.0001.

Forty-six miRNAs were deregulated (*P* < 0.05) in a comparison between HoV-PTC and *BRAF* p.K601E FV-PTC cases (regardless of other *BRAF* mutations; **Figure 2A**). **Table 2** shows 14 deregulated miRNAs with a *P*-value <0.01; seven of these were upregulated in HoV-PTC, while the remaining seven were downregulated.

**Table 2:** List of miRNAs deregulated in the comparison of HoV-PTCs vs. p.K601E-PTCs (*P* < 0.01).

MicroRNA_ID	HoV-PTCs (Relative expression)	*BRAF* p.K601E FV-PTC (Relative expression)	*P*-value
hsa-miR-127-3p	38.97	20.19	0.00008
hsa-miR-146b-5p	1,087.18	208.17	0.00017
hsa-miR-21-5p	3,828.23	920.68	0.00080
hsa-miR-205-5p	83.43	21.71	0.00154
hsa-miR-3065-5p	35.89	69.93	0.00176
hsa-miR-379-5p	24.53	20	0.00387
hsa-miR-3151-5p	159.86	583	0.00481
hsa-miR-4454+hsa-miR-7975	27,491.2	61,250.01	0.00670
hsa-miR-148b-3p	133.24	281.28	0.00718
hsa-miR-1285-5p	47.04	142.18	0.00728
hsa-miR-376c-3p	25.61	20	0.00919
hsa-miR-27a-3p	24.65	20	0.00934
hsa-miR-30c-5p	190.54	408.81	0.01074
hsa-miR-5196-3p+hsa-miR-6732-3p	96.18	210.32	0.01387

HoV-PTC, hobnail papillary thyroid carcinoma; BRAF p.K601E FV-PTC, BRAF K601E mutated papillary thyroid carcinoma.

Dividing the cohort of HoV-PTC cases based on the results of *BRAF* p.V600E analysis, we noted that a set of 127 miRNAs was deregulated between the two subgroups (*P* < 0.01). If we compare only the HoV-PTC p.V600E mutated subgroup with *BRAF* p.K601E FV-PTC cases, a large set of 324 miRNAs were significantly deregulated.

When HoV-PTC and CV-PTC samples were compared, 40 miRNAs were found to be downregulated in HoV-PTC patients, while 14 were overexpressed (*P* < 0.05). **Table 3** shows deregulated miRNAs with a *P*-value <0.01.

**Table 3:** List of miRNAs deregulated in the comparison of HoV-PTCs vs. CV-PTCs.

MicroRNA_ID	HoV-PTCs (Relative expression)	CV-PTCs (Relative expression)	*P*-value
hsa-miR-330-3p	2.06	6.74	7.1E-06
hsa-miR-3180-5p	1.67	3.59	0.00188
hsa-miR-519b-5p+hsa-miR-519c-5p+hsa-miR-523-5p+hsa-miR-518e-5p+hsa-miR-522-5p+hsa-miR-519a-5p	1.94	3.96	0.00524
hsa-miR-4286	4,923.05	1,195.28	0.00776
hsa-miR-539-5p	20.73	9.54	0.01012
hsa-miR-486-3p	2.04	4.21	0.01065
hsa-miR-556-5p	2.01	3.66	0.01075
hsa-miR-323b-3p	3.14	6.99	0.01329
hsa-miR-875-3p	1.57	3.32	0.01392
hsa-miR-503-5p	66.96	25.52	0.01489

HoV-PTC, hobnail papillary thyroid carcinoma; CV-PTCs, classic papillary thyroid carcinoma.

When the analysis focused on *BRAF* status, 14 miRNAs were deregulated in p.V600E HoV-PTC compared to p.V600E CV-PTC cases (seven were downregulated and seven upregulated; *P* < 0.01). Instead, comparing p.V600E CV-PTC to HoV-PTC wild type (wt), 43 miRNAs showed statistically significant differences in expression levels.

When the analysis compared *BRAF* p.K601E FV-PTC and CV-PTC samples, a set of 153 miRNAs were found to be deregulated (*P* < 0.05); in particular, only 14 were overexpressed in *BRAF* p.K601E FV-PTC patients, while the others were downregulated. **Table 4** shows the deregulated miRNAs with a *P*-value <0.01.

**Table 4:** List of miRNAs deregulated in the comparison of *BRAF* p.K601E FV-PTC vs. CV-PTCs (*P* < 0.01).

MicroRNA_ID	*BRAF* p.K601E FV-PTC (Relative expression)	CV-PTCs (Relative expression)	*P*-value
hsa-miR-675-5p	1.2	4.07	0.000003
hsa-miR-1250-5p	1.53	5.06	0.000004
hsa-miR-589-5p	1.81	4.11	0.00011
hsa-miR-433-5p	2.03	6.6	0.00016
hsa-miR-381-3p	2.42	6.15	0.00044
hsa-miR-92b-3p	3.01	10.72	0.00047
hsa-miR-4755-5p	1.63	5.28	0.00071
hsa-miR-34c-3p	2.88	8.97	0.00135
hsa-miR-208a-3p	1.19	3.4	0.00136
hsa-miR-376a-2-5p	2.09	5.37	0.00162
hsa-miR-1296-5p	1.32	3.52	0.00169
hsa-miR-640	2.59	7.21	0.00200
hsa-miR-129-2-3p	3.11	6.98	0.00203
hsa-miR-1193	2.09	6.06	0.00230
hsa-miR-196a-5p	3.12	13.93	0.00230
hsa-miR-526a+hsa-miR-518c-5p+hsa-miR-518d-5p	2.8	10.04	0.00319
hsa-miR-2053	1.95	7.53	0.00323
hsa-miR-330-3p	2.38	6.74	0.00426
hsa-miR-1298-5p	2.63	8.15	0.00547
hsa-miR-608	2.4	6.61	0.00573
hsa-miR-889-3p	2.13	4.86	0.00650
hsa-miR-1226-3p	2.13	6.16	0.00654
hsa-miR-561-5p	14.97	5.85	0.00665
hsa-miR-4454+hsa-miR-7975	51,688.68	16,905.97	0.00673
hsa-miR-488-3p	1.91	4.74	0.00697
hsa-miR-1245b-5p	1.25	4.73	0.00768
hsa-miR-1273c	1.73	3.43	0.00776
hsa-miR-875-3p	1.38	3.32	0.00836
hsa-miR-328-5p	1.57	4.29	0.00855
hsa-miR-941	2.63	6.29	0.00905
hsa-miR-542-3p	2.14	9.32	0.00954
hsa-miR-215-5p	1.6	3.72	0.00993
hsa-miR-197-5p	5.98	15.91	0.01000

BRAF p.K601E FV-PTC, BRAF K601E mutated papillary thyroid carcinoma; CV-PTC, classic papillary thyroid carcinoma.

Dividing the cohort of CV-PTC cases on the basis of a *BRAF* p.V600E analysis, we noted that a set of 65 miRNAs was deregulated between the two subgroups. In particular, only three miRNAs were upregulated in a *BRAF* p.K601E FV-PTC compared to p.V600E CV-PTC cohort, while all the others were downregulated (*P* < 0.05).

### Validation of MiRNAs Found to Be Deregulated in the Investigated Cohorts

For the validation of miRNAs associated with the histological and molecular features of PTC, we focused on miRNAs upregulated in a comparison of hobnail variant of PTC vs. p.K601E mutation and the classic variant of PTC based on the following observations: (1) since the hobnail variant is associated with a worse prognosis than those of patients with the classic variant and *BRAF* p.K601E mutation, miRNAs overexpressed in these samples might be important in determining the biological behavior of disease; (2) overexpressed rather than silenced miRNAs might be easier to detect in tissues and biological fluids. The expression of the three miRNAs (miR-21-5p, miR-146b-5p, and miR-205) deregulated in HoV-PTC vs. *BRAF* p.K601E FV-PTC and CV-PTC detected in the cohorts by nCounter^®^ was confirmed in the same cohorts (n = 35) using qRT–PCR. All three miRNAs were shown to be significantly upregulated in HoV-PTC patients (**Table 5**). Moreover, when the three miRNAs were analyzed in the subgroups divided for the *BRAF* p.V600E mutation, two of them (miR-146b and miR-21) were found to be overexpressed in all *BRAF* wt samples, while miR-205 was overexpressed in CV-PTC wt vs. CV-PTC p.V600E but downregulated in HoV-PTC wt vs. HoV-PTC p.V600E (**Figure 2B**).

**Table 5:** MiRNA expression determined by qRT–PCR in investigated cohort.

Variable	Compared cohorts	*P*-value
**miR-21-5p**	HoV-PTC vs. *BRAF* p.K601E FV-PTC	0.0059
	HoV-PTC vs. CV-PTC	0.0081
	*BRAF* p.K601E FV-PTC vs. CV-PTC	n.s.
	p.V600E HoV-PTC vs. *BRAF* p.K601E FV-PTC	0.06
	wt HoV-PTC vs. *BRAF* p.K601E FV-PTC	0.0001
	wt HoV-PTC vs. p.V600E HoV-PTC	0.0038
**miR-146b-5p**	HoV-PTC vs. *BRAF* p.K601E FV-PTC	0.0056
	HoV-PTC vs. CV-PTC	n.s.
	*BRAF* p.K601E FV-PTC vs. CV-PTC	0.0132
**miR-205-5p**	HoV-PTC vs. *BRAF* p.K601E FV-PTC	0.0098
	HoV-PTC vs. CV-PTC	n.s.
	*BRAF* p.K601E FV-PTC vs. CV-PTC	n.s.

HoV-PTC, hobnail papillary thyroid carcinoma; BRAF p.K601E FV-PTC, BRAF K601E mutated papillary thyroid carcinoma; CV-PTC, classic papillary thyroid carcinoma; n.s., not significant.

### MiR-21 and MiR-205 *In Situ* Hybridization Results

**Figure 3:**
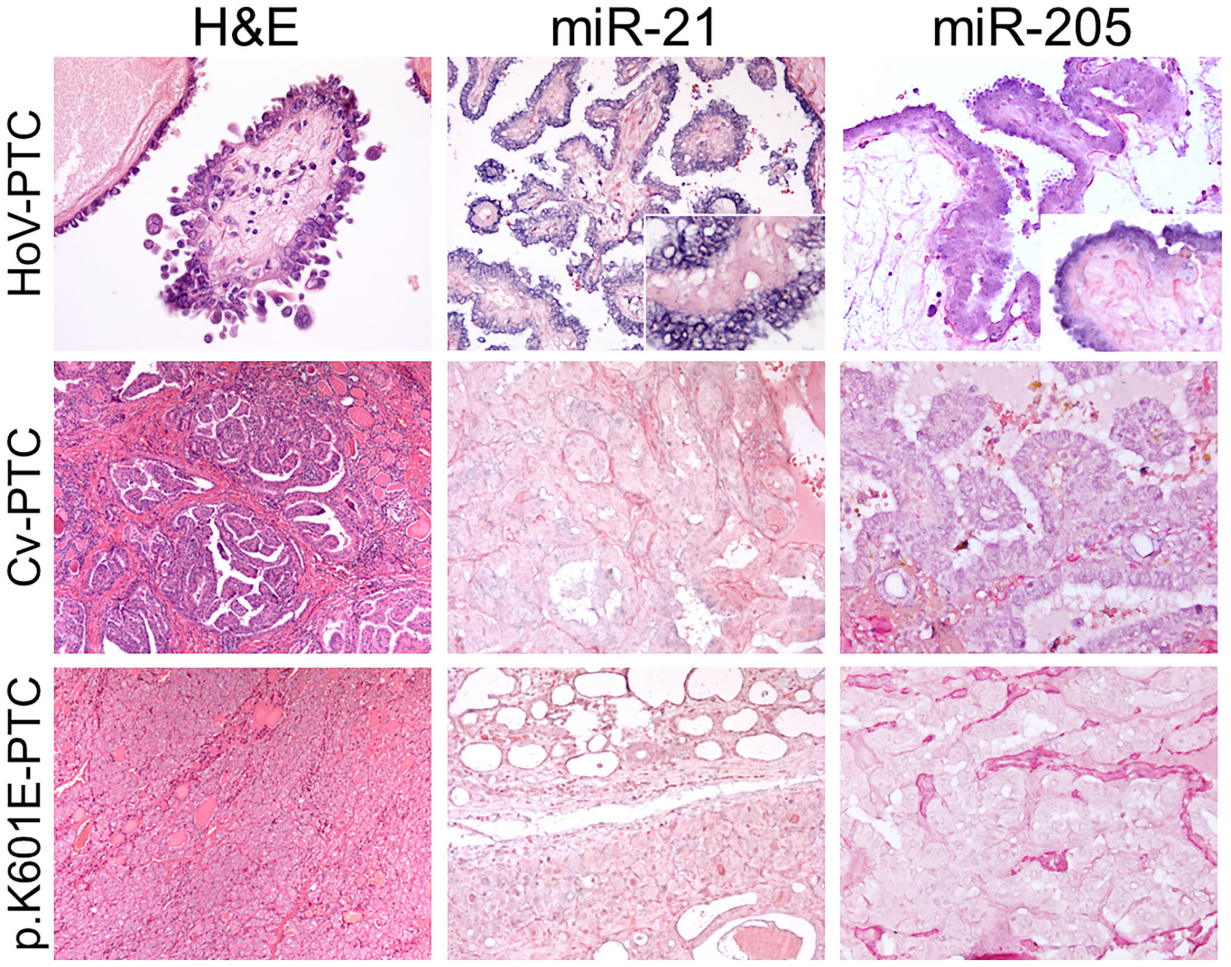
MiR-21 and miR-205 *in situ* hybridization in HoV-PTC, CV-PTC, and *BRAF* p.K601E FV-PTC. The first column represents hobnail papillary thyroid carcinoma (HoV-PTC), classic variant (CV)-PTC, and *BRAF* p.K601E FV-PTC tissues stained with hematoxylin and eosin. The expression of miRs was detectable as a grainy blue cytoplasmic stain. Significant overexpression of miR-21 and miR-205 (score 2) was observed in HoV. In CV-PTC samples, faint miR-21 and miR-205 (score 1) staining was observed. Positive cytoplasmic staining for miR-21 and miR-205 in *BRAF* p.K601E FV-PTC tissue compared with adjacent normal thyroid tissue was not observed (×200 and ×100 original magnification).

To further confirm nCounter^®^ and qRT–PCR findings, miR-21 and miR-205 expressions were investigated in all samples (n = 35) by ISH analysis. The expression of miR-21 and miR-205 was identifiable as a granular blue cytoplasmic stain that was scored according to a three-tiered scale (score 0, no cytoplasmic staining; score 1, faint cytoplasmic staining; score 2, strong cytoplasmic staining). In HoV-PTC, miR-21 was significantly overexpressed: all (9/9) wt HoV-PTC cases were given a score of 2; 63% (5/8) of p.V600E HoV-PTC cases were scored 2, and 37% (3/8) were scored 1 (by comparison with adjacent normal thyroid tissue). Instead, all *BRAF* p.K601E FV-PTC cases were given a score of 0; all (5/5) wt CV-PTC cases and 75% (3/4) of p.V600E CV-PTC cases were given a score of 1, while 25% (1/4) of p.V600E CV-PTC cases were given a score of 2 (by comparison with adjacent normal thyroid tissue; **Figure 3**). Also, miR-205 was significantly overexpressed in HoV-PTC cases (14/17 scored 2); instead, all *BRAF* p.K601E FV-PTC cases were negative (score 0) for the stain. CV-PTC cases were principally given a score of 1 (7/9; **Figure 3**).

## Discussion

PTC is a heterogeneous neoplasia comprising several histological variants that are associated with specific mutational profiles with distinct clinical and prognostic features. Therefore, novel markers are being studied in order to better characterize these particular subtypes of PTC and to achieve targeted follow-up and treatments.

For this purpose, we used an innovative method that can simultaneously analyze a wide range of miRNAs, generating a “miRnoma” of the cancer. Researchers have demonstrated the value of the automated platform, NanoString Technologies nCounter^®^, in several publications, with results derived from a variety of tissues and in different biological settings, including cancer development, neurobiology, and immunology. Through NanoString, it is possible to analyze up to 800 miRNAs for each single sample—thanks to a hybridization system using fluorescent barcodes. The main advantages of using this platform concern the high sensitivity and reproducibility of the results even with samples that are usually difficult to analyze, such as formalin-fixed paraffin-embedded tissues (23).

In our series, we decided to compare forms of PTC with completely different clinical behavior, obviously considering them from a histological but also a molecular point of view, taking into consideration the particular variant with the p.K601E BRAF mutation. The latter is identified almost exclusively in the follicular histological variant of PTC, as we reported in our series, and usually also distinguishes capsulated forms of the follicular variant, therefore with even less aggressive characteristics (24). We preferred to go beyond the concept of the histological variant of PTC by including in the categorization also molecular information to further stratify the neoplastic behavior.

Our analysis revealed significant differences in terms of a miRNA expression profile between the different cohorts analyzed. In particular, 46 miRNAs were deregulated in a comparison of HoV-PTCs and *BRAF* p.K601E FV-PTCs. A set of 153 miRNAs were found to be deregulated between *BRAF* p.K601E FV-PTC and CV-PTC samples. And in a comparison between HoV-PTC and CV-PTC samples, 54 miRNAs were deregulated (*P* < 0.05). After a subdivision of the two cohorts (HoV-PTC and CV-PTC) according to molecular results for *BRAF* p.V600E, a statistically significant difference was found in the expression profile of the same cohorts (for example, p.V600E HoV-PTC vs. wt HoV-PTC) and between different cohorts (for example, p.V600E HoV-PTC vs. p.V600E CV-PTC). Among the more deregulated miRNAs, three miRNAs primarily emerged: miR-21-5p, miR 146b-5p, and miR-205-5p. Validation analysis (RT–PCR and ISH) confirmed these results.

The miRNA, miR-21-5p, is a crucial oncomir that has been found to be linked to proliferation, apoptosis, invasion, and migration of malignant cell lines (25). Thus, miR-21-5p plays a fundamental role in malignant behavior and transformation. In thyroid carcinogenesis and specifically in PTC, the deregulation of miR-21-5p has been demonstrated (26). The role of miR-21-5p lies in its specific targeting of the three prime UTR (3′-UTR) of *PDCD4*, which negatively regulates protein expression and is a well-known tumor suppressor. The importance of this molecular pathway (which might not necessarily be the sole one) in regulation by miR-21-5p is supported by the finding that miR-21-5p upregulation consistently parallels programmed cell death 4 protein downregulation (27).

The second miRNA, miR-146-5p, has a regulatory role in several tumors by acting on invasiveness and metastasis. For example, it has been shown that breast cancer metastasis-suppressor 1 (BRMS1) affects mir-146 (28). Nuclear factor-κB (NF-κB), which is regulated by BRMS1 and mir-146, is involved in the development of cancer by influencing the proliferative and antiapoptotic signals of thyroid carcinomas (29). NF-κB is implicated in anaplastic thyroid carcinoma through the overexpression of mir-146a. It has been shown that the upregulation of mir-146b reduces the growth of primary brain tumors as gliomas. Several studies have shown that transforming growth factor (TGF)-β1 regulates specific miRNAs, such as mir-146b-5p, in normal cells and cancer. Because TGF-β1 also regulates epithelial–mesenchymal transition in PTC, this growth factor has important roles in the expression of specific miRNAs for the progression of thyroid cancer (30–32). Inactivating miR-146b has been considered as a promising strategy also in thyroid cancer therapy. For example, Santa-Inez et al. (33) targeted miR-146b in an experimental model conducted on anaplastic thyroid cancer cell line using Clustered Regularly Interspaced Short Palindromic Repeats / Cas9n (CRISPR/Cas9n) editing system. Also in follicular thyroid cancer (FTC), miR-146b was found to be associated with the process of cancerization and increased aggressiveness of the neoplasm. In particular, Knyazeva et al. (34) analyzed a series of 84 follicular lesions with different histological diagnosis and found a correlation with miR-146b dysregulation and follicular cell malignant transformation and follicular thyroid cancer progression. miR-146b has also been proposed as a circulating marker of PTC, both diagnostically and prognostically, but future studies will be necessary for this application (35).

In particular, the overexpression of miR-205-5p in the hobnail variant vs. p.K601E-PTC is noteworthy. Importantly, miR-205 is a driver miRNA whose deregulation may induce cancer development and progression (36, 37). Moreover, miR-205 has a double function: i) it can play a role as an oncogene in several cancers (for example, lung, bladder, cervical, and head/neck malignancies); and ii) it can be downregulated in breast cancer, prostatic cancer, and glioma (38, 39). The only previous study that investigated miR-205 expression in thyroid neoplasia suggested that miR-205 overexpression might have a potential tumor suppressive role, which could be exerted through miR-205 binding to 3-UTR of vascular endothelial growth factor A (VEGFA) protein in thyroid cancer. That the overexpression of this miRNA has been found in cases of aggressive variant PTC, such as hobnail-PTC, underlies the hypothesis that the regulatory mechanism of this miRNA is more complex and, therefore, requires further investigation to be clearly understood.

In conclusion, PTC is a heterogeneous neoplasm from both morphological and molecular points of view. The present study highlights this by demonstrating how the different histological variants and specific *BRAF* mutations, which are associated with a different prognosis, are characterized by different miRNA expression profiles. The miRNAs, miR-21-5p, miR-146b-5p, and miR-205-5p, were the most significantly overexpressed miRNAs in the analyzed cohorts. In light of these data, although results are preliminary, these markers appear to play a role in PTC carcinogenesis and, in particular, seem to correlate with its different biological behavior. This is a seminal study that will be deepened considering the most relevant emerged miRNAs in large series for each of the groups. One of the limitations of this study is the low number of analyzed samples mainly due to the rarity of the categories considered such as the p.K601E *BRAF* mutation and the hobnail variant. Further studies are necessary in order to validate these results in a wider population-based cohort and to understand the exact mechanism of action of these miRNAs.

## Data Availability Statement

The original contributions presented in the study are included in the article/supplementary material. Further inquiries can be directed to the corresponding author.

## Ethics Statement

The studies involving human participants were reviewed and approved by the University Hospital of Padua Ethics Committee. Written informed consent for participation was not required for this study in accordance with the national legislation and the institutional requirements.

## Author Contributions

FG designed the study, performed the research, analyzed the data, and wrote the article. SC, IMB, MF, NV, MR, FV, ADT, CM, and GP designed the study and performed the research. MS, JCH, LB, GM, MG, LC, and SB contributed to the acquisition of clinical samples and data and to the statistical analysis. All authors contributed to the article and approved the submitted version.

## Funding

This research did not receive any specific grant from any funding agency in the public, commercial, or not-for-profit sectors. MF is supported by a grant from the Italian Health Ministry/Veneto region research program NET-2016–02363853 and Fondazione AIRC under 5 per mille, grant ID 22759.

## Conflict of Interest

The authors declare that the research was conducted in the absence of any commercial or financial relationships that could be construed as a potential conflict of interest.

## Publisher’s Note

All claims expressed in this article are solely those of the authors and do not necessarily represent those of their affiliated organizations, or those of the publisher, the editors and the reviewers. Any product that may be evaluated in this article, or claim that may be made by its manufacturer, is not guaranteed or endorsed by the publisher.
